# Neutrophil to Lymphocyte Ratio Predicts Outcome of Stroke by Cervicocranial Arterial Dissection

**DOI:** 10.3389/fmed.2020.598055

**Published:** 2020-11-27

**Authors:** Guangbi Sun, Yi Yang, Zhiguo Chen, Le Yang, Shanshan Diao, Shicun Huang, Yiqing Wang, Yiting Wang, Baoliang Sun, Xia Yuan, Xingshun Xu

**Affiliations:** ^1^Department of Neurology, The First Affiliated Hospital of Soochow University, Suzhou, China; ^2^School of Public Health, Fujian Medical University, Fuzhou, China; ^3^Department of Neurology, The Second Affiliated Hospital, Shandong Academy of Medical Sciences, Shandong First Medical University, Taian, China; ^4^The Institute of Neuroscience, Soochow University, Suzhou, China

**Keywords:** cervicocranial arterial dissection, acute ischemic stroke, inflammation, neutrophil to lymphocyte ratio, 3-month outcome

## Abstract

**Background and Purpose:** Neutrophil to lymphocyte ratio (NLR) is positively associated with poor prognosis in patients with cerebral infarction. The goal of this prospective study is to explore the predictive value of NLR in patients with acute ischemic stroke (AIS) caused by cervicocranial arterial dissection (CCAD).

**Methods:** Ninety-nine patients with AIS caused by CCAD met criteria for inclusion and exclusion were selected for this study. We collected baseline data on the admission including NLR. The primary poor outcome was major disability (modified Rankin Scale score ≥ 3) or death at 3 months after AIS.

**Results:** A total of 20 (20.2%) patients had a poor outcome at 3 months after AIS. According to the 3-month outcome, the patients were divided into two groups and univariate and multivariable analyses were conducted. Among the risk factors, elevated NLR levels were independently associated with 3-month poor outcomes. Further, we made the ROC curve to evaluate the predictive value of NLR level on prognosis. The area under the curve was 0.79 and a cut-off value of NLR was 2.97 for differentiating the poor outcome. We divided patients into groups according to the cut-off value. Patients with high NLR have a higher risk of poor outcome than those with low NLR (*P* < 0.05).

**Conclusion:** As an inflammatory marker, elevated NLR levels were associated with 3-month poor outcome in AIS caused by CCAD.

## Introduction

Arterial dissection is the angiopathy that blood flow enters the artery wall and causes vascular wall tissue dissect. Arterial dissection leads to various pathological changes, such as the stenosis or occlusion of the lumen and dissecting aneurysm of the artery (1). With the development of imaging techniques and clinical knowledge, incidence of cervicocranial arterial dissection (CCAD) is increasing. It has become a common cause of stroke in young patients, in whom it accounts for 10–25% (2). The etiology of CCAD is still unclear. Trauma and connective tissue diseases can only explain partial dissection (3). Inflammation is known to be involved in the development of a variety of vascular diseases (4–7). Recently, infections have been reported to be associated with the occurrence and pathogenesis of CCAD (8). In previous studies on AIS, inflammation and immune response are also important parts of pathophysiology of stroke (9–11). Therefore, we speculated inflammatory response is important in AIS by CCAD.

The neutrophil to lymphocyte ratio (NLR), as a simple parameter of innate (neutrophil), and adaptive (lymphocyte) immune response, is easy to obtain from peripheral blood (12). Recent studies show NLR can predict the prognosis and mortality of patients with aortic dissection or cerebral infarction (5, 13, 14). Qun et al. (15) demonstrated that high NLR was highly correlated with the 3-month poor outcome of AIS. Clinical values of NLR in prognoses of patients with AIS caused by CCAD have not been fully explored. Therefore, we conducted a comprehensive analysis of those patients to explore the prognostic value of NLR on the 3-month outcome.

## Materials and Methods

### Study Population

Patients with AIS by CCAD in the First Affiliated Hospital of Soochow University from April 2014 to October 2019 were selected as the research subjects. Data were collected from electronic patient records and administrative databases used for quality improvement.

CCAD was initially diagnosed by computed tomography angiography (CTA), and was further confirmed by digital subtraction angiography (DSA) or high-resolution magnetic resonance imaging (HR-MRI); in addition, thickened vascular intima and atherosclerotic plaque formation were excluded (16). All cases of AIS were confirmed by MRI. The diagnosis of CCAD was made according the previous description (17). The diagnostic criteria included the following factors: clinical symptoms such as neck pain, edema, and signs of Horner's syndrome; the disruption of normal arterial wall on CTA or DSA imaging including stenosis, intimal flap, false lumen, mural thrombus, and pseudoaneurysm; the exclusion of vessel hypoplasia, pseudodissection, and the signs of atherosclerosis such as vessel calcification (17). We confirmed the relationship between CCAD and AIS that the dissected artery was the only responsible vessel and the only cause of AIS by HR-MRI or CTA imaging. In addition, cardiogenic stroke was excluded in all patients by cardiac examination.

Exclusion criteria were as follows: (1) Patients with AIS admitted more than 48 h; (2) Patients had a history of infection within 2 weeks before admission that was defined as fever (T ≥ 38°C) and at least one other typical symptoms (cough, rhinitis, hoarseness, sneezing, or vomiting); (3) Patients had a history of cancer, chronic inflammation, hematological diseases, autoimmune diseases, or treatment with immunosuppressive agents; (4) Patients had a stroke history within 6 months or the modified Rankin scale (mRS) > 0 before the onset; (5) Patients did not complete a blood count within 24 h of admission; (6) There was no evidence of AIS at this admission; (7) patients with iatrogenic and traumatic dissections. The diagnosis of CCAD was made by two senior imaging doctors.

### Clinical Information Collection

We collected baseline data including gender, age, history of trauma, history of head and neck pain, cerebral vascular risk factors such as hypertension, and diabetes. Peripheral venous blood samples were collected on the morning of the second day after admission with an overnight fasting.

### Evaluation of 3-Month Outcome

Modified Rankin Scale (mRS) was used to evaluate the 3-month outcome after the onset of AIS. The primary outcome was death or major disability at 3 months after AIS. other outcomes were stroke recurrence and hemodynamics of the diseased vessels. The poor outcome was defined as the mRS ≥ 3.

### Statistical Analysis

Continuous variables were analyzed as mean and standard deviation or the median and interquartile range while categorical variables were analyzed as frequency and percentage, properly. The differences among continuous variables were analyzed by the Student's *t-*test or the Mann-Whitney *U-*test while differences among categorical variables were assessed by the Chi-square test. Logistic regression analysis was used to find risk factors associated with poor prognosis in patients with AIS caused by CCAD after adjusting for other variables selected from univariate analyses. Receiver operating curves (ROC) were used to evaluate the predictive value of NLR level and to establish optimal cut-off values of NLR correlated with poor outcome. Statistical analysis was performed in SPSS 25.0. A value of *P* < 0.05 was considered statistically significant.

## Results

### Study Population and Baseline Characteristics

A total of 168 patients with CCAD were admitted between April 2014 and October 2019 in the First Affiliated Hospital of Soochow University (Suzhou city, China). The CCAD mainly showed dual-chamber sign, line-like sign, endometrial flap sign, bead sign, or rat tail sign in CTA or DSA examination. HR-MRI revealed signs of hematoma, aneurysm-like dilatation, or double cavity with true cavity stenosis in the dissection. Among these patients, 69 patients were excluded according to the exclusion criteria, and 99 patients met the study criteria. Patient baseline characteristics were shown in Table 1. The average age of all patients was 47.72 ± 11.94; 79 (79.8%) were male and the ratio of male to female was about 4:1. 17 (17.2%) patients had a smoking history; 43 (43.4%) had a hypertension history; 11 (11.1%) had type 2 diabetes; 11 (11.1%) had a stroke history or TIA; and 18 (18.2%) had a history of trauma. White blood cell (WBC) was 8.91 ± 2.88 × 10^9^ /L; neutrophil count was 5.54 (4.08, 7.88) × 10^9^/L, and lymphocyte count was 1.64 (1.27, 2.09) × 10^9^ /L; NLR was 3.21 (2.44, 4.91). All of our patients received anticoagulant or antiplatelet therapy and five patients (5.05%) stopped medication after intracranial/gastrointestinal hemorrhage during treatment. Among them, 20 (20.2%) patients had a poor outcome at 3 months after AIS; 3 (3%) patients had recurrent ischemic stroke, and 58 (58.6%) patients of ultrasound showed improved hemodynamics of diseased vessels.

**Table 1:** The clinical characteristics of patients with AIS caused by CCAD.

**Characteristics**	**Patients (*n =* 99)**
**Demographics**	
Age in years, mean ± SD	47.72 ± 11.94
Male, *n* (%)	79 (79.80)
Smoking, *n* (%)	17 (17.20)
Drinking, *n* (%)	7 (7.10)
**Medical history**	
History of trauma, *n* (%)	18 (18.20)
History of head and neck pain, *n* (%)	16 (16.20)
Hypertension, *n* (%)	43 (43.40)
Diabetes, *n* (%)	11 (11.10)
CHD, *n* (%)	1 (1.00)
History of stoke or TIA, *n* (%)	11 (11.10)
**Clinical features**	
Patients with vascular occlusion, *n* (%)	59 (59.60)
SBP in mmHg, mean ± SD	132.03 ± 16.80
DBP in mmHg, mean ± SD	80.60 ± 12.69
FGB in mmol/l, median (IQR)	5.01 (4.49, 6.01)
TC in mmol/l, median (IQR)	3.83 (3.23, 4.57)
TG in mmol/l, median (IQR)	1.23 (0.95, 1.73)
HDL in mmol/l, median (IQR)	1.01 (0.87, 1.23)
LDL in mmol/l, median (IQR)	2.23 (1.68, 2.85)
WBC in × 10^9^/l, mean ± SD	8.91 ± 2.88
NLR, median (IQR)	3.21 (2.44, 4.91)
N in × 10^9^/l, median (IQR)	5.54 (4.08, 7.88)
L in × 10^9^/l, median (IQR)	1.64 (1.27, 2.09)
CRP, median (IQR)	2.72 (0.73, 9.22)
NIHSS at admission, median (IQR)	4.00 (2.00, 10.00)
NIHSS at discharge, median (IQR)	2.00 (1.00, 6.00)
**Primary outcome: major disability (mRS score 3-6)**	
3-month mRS, median (IQR)	1.00 (0.00, 2.00)
3-month poor outcome, *n* (%)	20 (20.20)
**Other outcomes**	
Hemorrhage, *n* (%)	5 (5.05)
Recurrent ischemic stroke, *n* (%)	3 (3.00)
Improved hemodynamics of diseased vessels, *n* (%)	58 (58.60)

### Risk Factors Associated With Poor Outcome in Patients With AIS Caused CCAD

According to the outcome after a 3-month follow-up, the patients were divided into two groups: the good outcome group with 79 patients (mRS < 3) and the poor outcome group with 20 patients (mRS ≥ 3). Statistical analysis indicated that there were significant differences on FGB, WBC, NLR, neutrophil count, C-reactive protein, NIHSS at admission and NIHSS score at discharge, 3-month mRS between two groups (*P* < 0.05); However, there was no difference on age, gender, history of trauma, hypertension, diabetes, CHD, smoking, or drinking, TC, TG, HDL, LDL, neutrophil count, recurrent ischemic stroke, improved hemodynamics of diseased vessels, hemorrhage, and other factors between two groups (*P* > 0.05, Table 2).

**Table 2:** Clinical and laboratory findings in patients with poor and good outcome.

**Characteristics**	**mRS ≥ 3 (*N =* 20)**	**mRS < 3 (*N* = 79)**	***P*-value**
**Demographics**			
Age in years, mean ± SD	51.35 ± 9.58	46.80 ± 12.35	0.13
Male, *n* (%)	17 (85.00)	62 (78.50)	0.76
Smoking, *n* (%)	2 (10.00)	15 (19.0)	0.51
Drinking, *n* (%)	1 (5.00)	6 (7.60)	1.00
**Medical history**			
History of trauma, *n* (%)	3 (15.00)	15 (19.00)	1.00
History of head and neck pain, *n* (%)	1 (5.00)	15 (19.00)	0.18
Diabetes, *n* (%)	4 (20.00)	7 (8.90)	0.23
Hypertension, *n* (%)	10 (50.00)	33 (41.80)	0.62
CHD, *n* (%)	0 (0.00)	1 (1.30)	1.00
History of stroke or TIA, *n* (%)	1 (5.00)	10 (12.70)	0.45
**Clinical features**			
Patients with vascular occlusion, *n* (%)	15 (75.00)	44 (55.70)	0.13
SBP in mmHg, median (IQR)	138.50 (119.25, 150.00)	130.00 (121.00, 139.00)	0.32
DBP in mmHg, median (IQR)	80.50 (67.75, 90.00)	80.00 (72.00, 90.00)	0.96
FGB in mmol/l, median (IQR)	6.02 (5.13, 7.87)	4.88 (4.46, 5.50)	0.01
TC in mmol/l, median (IQR)	4.04 (3.36, 4.72)	3.77 (3.20, 4.50)	0.20
TG in mmol/l, median (IQR)	1.36 (1.02, 1.75)	1.19 (0.94, 1.68)	0.44
HDL in mmol/l, median (IQR)	1.05 (0.91, 1.32)	0.99 (0.87, 1.18)	0.43
LDL in mmol/l, median (IQR)	2.48 (1.90, 2.83)	2.13 (1.65, 2.91)	0.22
WBC in × 10^9^/l, mean ± SD	11.12 ± 3.21	8.35 ± 2.51	<0.001
NLR, median (IQR)	5.26 (3.25, 7.81)	2.90 (2.16, 4.23)	<0.001
N in × 10^9^/l, median (IQR)	8.62 (5.91, 10.87)	5.08 (3.83, 6.66)	<0.001
L in × 10^9^/l, median (IQR)	1.56 (1.05, 1.88)	1.64 (1.28, 2.20)	0.20
CRP, median (IQR)	11.27 (4.10, 14.04)	2.07 (0.68, 6.05)	0.002
NIHSS at admission, median (IQR)	16.00 (12.00, 17.50)	3.00 (2.00, 7.00)	<0.001
NIHSS at discharge, median (IQR)	10.00 (8.00, 11.75)	2.00 (1.00, 4.00)	<0.001
**Outcome**			
3-month mRS, median (IQR)	3.50 (3.00, 4.00)	1.00 (0.00, 1.00)	<0.001
Hemorrhage, *n* (%)	2 (10.00)	3 (3.80)	0.06
Recurrent ischemic stroke, *n* (%)	1 (5.00)	2 (2.50)	0.50
Improved hemodynamics of diseased vessels, *n* (%)	10 (50.00)	48 (60.80)	0.45

Binary logistic regression analysis was used to determine factors that were significantly associated with poor outcome at 3 months after AIS. After the factors that might potentially affect the outcome were adjusted, our results indicated that NLR (adjusted OR, 2.457; 95%CI, 1.096–5.508; *P* = 0.03), TG (adjusted OR, 10.015; 95%CI, 1.143–87.736; *P* = 0.04), WBC (adjusted OR, 1.794; 95%CI, 1.056–3.049; *P* = 0.03), age (adjusted OR, 1.258; 95%CI, 1.015–1.559; *P* = 0.04), ANC (adjusted OR, 2.919; 95%CI, 1.198–7.111; *P* = 0.02), and NIHSS (adjusted OR, 1.767; 95%CI, 1.234–2.529; *P* = 0.002) at admission were associated with 3-month poor outcome in the study. However, history of trauma, history of head and neck pain, smoking, type 2 diabetes, SBP, FGB, LDL, history of stroke or TIA and ALC showed no association with poor outcome (*P* > 0.05, Table 3).

**Table 3:** Binary logistic regression analysis predicting the poor outcome in patients with AIS caused by CCAD.

**Model**	**Independent variable**	**Adjusted OR**	**95% CI**	***P*-value**
Model 1	Age	1.092	0.951–1.255	0.212
(with NLR)	History of trauma	0.225	0.016–3.237	0.273
	History of head and neck pain	0.013	0.000–5.504	0.159
	Smoking	5.190	0.217–123.918	0.309
	Diabetes	0.605	0.011–32.534	0.805
	NIHSS at admission	1.712	1.268–2.310	<0.001
	SBP	0.997	0.942–1.054	0.912
	FGB	1.444	0.746–2.798	0.276
	LDL	0.431	0.144–1.291	0.133
	TG	10.015	1.143–87.736	0.037
	History of stroke or TIA	0.318	0.001–164.345	0.719
	NLR	2.457	1.096–5.508	0.029
	CRP	0.937	0.786–1.117	0.470
Model 2	Age	1.160	0.999–1.347	0.052
(with WBC)	History of trauma	0.065	0.002–2.124	0.124
	History of head and neck pain	0.094	0.002–5.167	0.247
	Smoking	2.518	0.163–39.006	0.509
	Diabetes	0.445	0.013–14.924	0.651
	NIHSS at admission	1.586	1.222–2.058	0.001
	SBP	1.000	0.946–1.058	0.991
	FGB	1.380	0.749–2.542	0.301
	LDL	0.532	0.177–1.602	0.262
	TG	4.383	0.861–22.310	0.075
	History of stroke or TIA	0.447	0.003–71.029	0.756
	WBC	1.794	1.056–3.049	0.031
	CRP	1.018	0.868–1.193	0.829
Model 3	Age	1.258	1.015–1.559	0.036
(with ANC)	History of trauma	0.025	0.000–1.382	0.072
	History of head and neck pain	0.039	0.000–6.445	0.213
	Smoking	5.620	0.229–137.640	0.290
	Diabetes	0.125	0.001–24.015	0.439
	NIHSS at admission	1.767	1.234–2.529	0.002
	SBP	1.014	0.952–1.079	0.672
	FGB	1.695	0.668–4.304	0.267
	LDL	0.274	0.061–1.224	0.090
	TG	13.744	1.093–172.838	0.042
	History of stroke or TIA	0.040	0.000–533.520	0.507
	ANC	2.919	1.198–7.111	0.018
	CRP	1.038	0.866–1.244	0.688
Model 4	Age	1.037	0.934–1.152	0.495
(with ALC)	History of trauma	0.646	0.052–7.961	0.733
	History of head and neck pain	0.119	0.002–7.372	0.312
	Smoking	1.171	0.101–13.603	0.899
	Diabetes	1.326	0.053–33.282	0.864
	NIHSS at admission	1.637	1.289–2.078	0.000
	SBP	0.997	0.947–1.049	0.902
	FGB	1.254	0.800–1.968	0.324
	LDL	0.602	0.237–1.527	0.285
	TG	3.649	0.656–20.295	0.139
	History of stroke or TIA	1.363	0.047–39.716	0.857
	ALC	0.350	0.071–1.739	0.199
	CRP	1.005	0.881–1.147	0.942

*ANC, absolute neutrophil count; ALC, absolute lymphocyte count; OR, odds ratio; CI, confidence interval*.

### NLR Was Associated With 3-Month Poor Outcome

**Figure 1:**
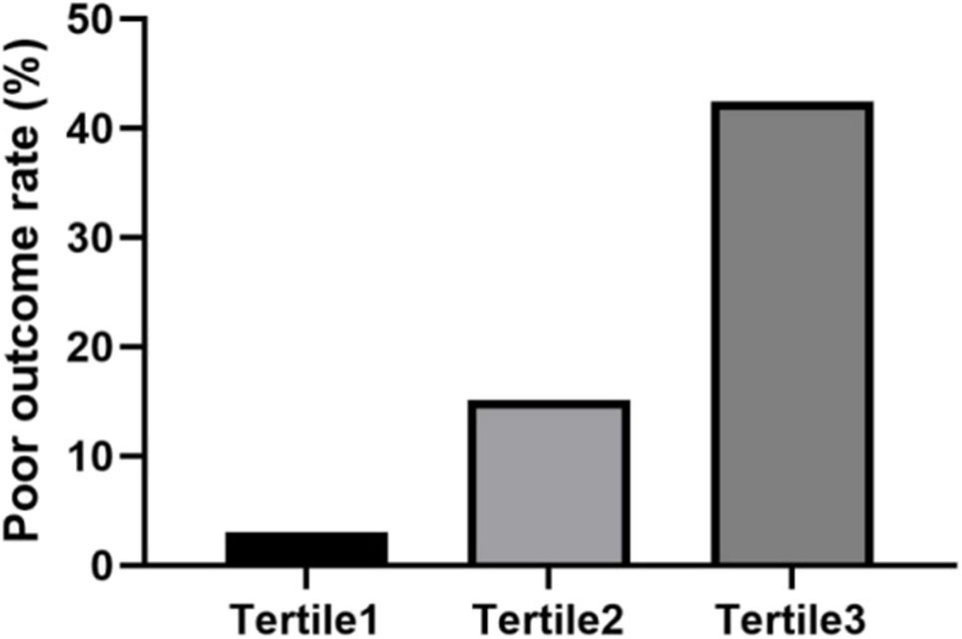
The percentage of patients with poor outcome was stratified by the tertile of NLR. Patients were divided into three groups according to the tertiles of NLR (NLR ≤ 2.59, 2.59 < NLR ≤ 4.23, NLR > 4.23). The poor outcome rate was calculated. The difference between each tertile was assessed by Jonckheere-Terpstra test (*P* < 0.001).

Since NLR is a simple and convenient biomarker to obtain and was validated in patients with AIS, we examined whether NLR was a more specific biomarker for 3-month outcome in patients with AIS caused by CCAD. According to the NLR value, the study population was divided into three tertiles, each containing 33 people. In the first tertile (NLR 2.05, 1.24–2.86), the poor outcome rate was 3%; in the second tertile (NLR 3.21, 2.42–4.00), the poor outcome rate was 15.2%; in the third tertile (NLR 6.15, 2.96–9.34), the poor outcome rate was 42.4% (Figure 1). We used the Jonckheere-Terpstra test to evaluate the relationship of the poor outcome rate and NLR and found that the difference on the poor outcome rate between each tertile was statistically significant (*P* < 0.001), indicating that high NLR level was associated with 3-month poor outcome.

**Figure 2:**
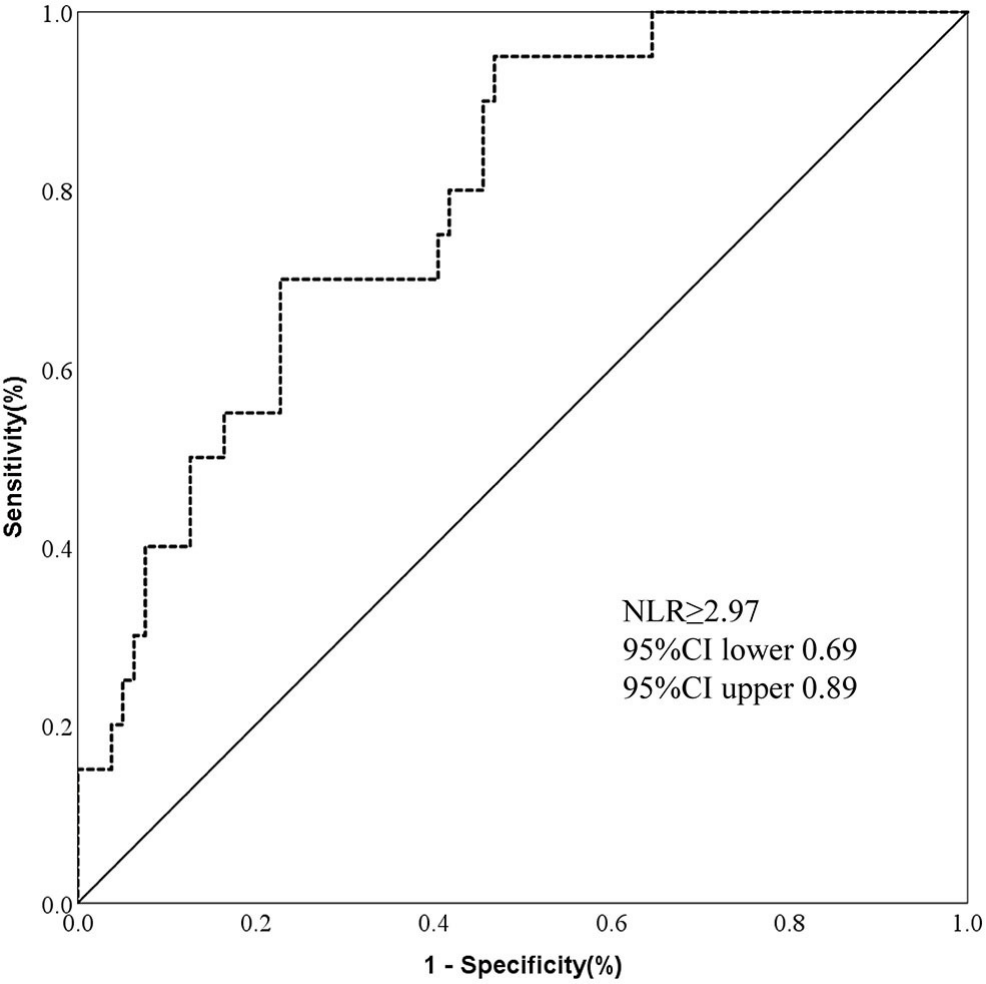
ROC showed predictive value of NLR for 3-month poor outcome in AIS by CCAD. [*n* = 99; sensitivity = 0.95; specificity = 0.53; NLR = 2.97; area under curve (AUC) = 0.79].

Further, we made the ROC curve to evaluate the predictive value of NLR level on prognosis. An NLR value of 2.97 was calculated as an optimal cut-off value to discriminate between good and poor outcome of patients with AIS caused by CCAD. The area under the curve was 0.79 (95%CI, 0.69–0.89). An NLR value of 2.97 as a cut-off value for differentiating the poor outcome with a sensitivity of 95% and a specificity of 53% (Figure 2).

### Patients With High NLR Have an Increased Risk of Poor Outcome

According to the cut-off point, the patients were divided into a high NLR group with 56 patients (≥3) and a low NLR group with 43 patients (<3). We found statistically significant differences on drinking, history of stroke or TIA, WBC, neutrophil count, lymphocyte count, C-reactive protein levels, NIHSS at admission, NIHSS score at discharge, and 3-month mRS between the two groups (*P* < 0.05). However, there was no statistically significant difference in other factors between the two groups (*P* > 0.05, Table 4). In the high NLR group, 19 patients (33.9%) had 3-month poor outcome, whereas in the low NLR group, 1 patient (2.3%) had 3-month poor outcome (Table 4). Compared with patients in the low NLR group, patients in the high NLR group had higher poor outcome rate (*P* < 0.01).

**Table 4:** Clinical and laboratory findings in patients with low and high NLR.

**Characteristics**	**NLR ≥ 2.97 (*N =* 56)**	**NLR < 2.97 (*N =* 43)**	***P-*value**
**Demographics**			
Age in years, mean ± SD	47.41 ± 10.88	48.12 ± 13.31	0.77
Male, *n* (%)	43.00 (76.80)	36.00 (83.70)	0.39
Smoking, *n* (%)	7.00 (12.50)	10.00 (23.30)	0.16
Drinking, *n* (%)	1.00 (1.80)	6.00 (14.00)	0.02
**Medical history**			
History of trauma, *n* (%)	10.00 (17.90)	8.00 (18.60)	0.92
History of head and neck pain, *n* (%)	10.00 (17.90)	6.00 (14.00)	0.60
Hypertension, *n* (%)	25.00 (44.60)	18.00 (41.90)	0.78
Diabetes, *n* (%)	7.00 (12.50)	4.00 (9.30)	0.62
CHD, *n* (%)	1.00 (1.80)	0.00 (0.00)	0.38
History of stroke or TIA, *n* (%)	3.00 (5.40)	8.00 (18.60)	0.04
**Clinical features**			
Patients with vascular occlusion, *n* (%)	32.00 (57.10)	27.00 (62.80)	0.57
SBP in mmHg, mean ± SD	134.34 ± 16.66	129.02 ± 16.69	0.12
DBP in mmHg, mean ± SD	81.84 ± 13.25	78.98 ±11.89	0.27
FGB in mmol/l, median (IQR)	5.32 (4.44, 6.66)	4.84 (4.61, 5.32)	0.41
TC in mmol/l, median (IQR)	3.88 (3.34, 4.71)	3.76 (3.16, 4.44)	0.33
TG in mmol/l, median (IQR)	1.31 (0.97, 1.67)	1.17 (0.95, 1.81)	0.42
HDL in mmol/l, median (IQR)	1.05 (0.90, 1.24)	0.95 (0.87, 1.18)	0.61
LDL in mmol/l, median (IQR)	2.26 (1.75, 2.97)	2.10 (1.56, 2.78)	0.14
WBC in × 10^9^/l, mean ± SD	10.06 ± 2.85	7.42 ± 2.15	<0.001
NLR, median (IQR)	4.73 (3.57, 6.68)	2.26 (1.71, 2.59)	<0.001
N in × 10^9^/l, median (IQR)	7.11 (5.55, 9.22)	4.08 (3.52, 4.96)	<0.001
L in × 10^9^/l, median (IQR)	1.49 (1.12, 1.84)	1.89 (1.52, 2.61)	<0.001
CRP, median (IQR)	5.61 (1.42, 13.26)	1.42 (0.49, 4.07)	0.01
NIHSS at admission, median (IQR)	8.00 (2.00, 14.00)	3.00 (2.00, 5.00)	<0.001
NIHSS at discharge, median (IQR)	5.00 (1.25, 8.75)	1.00 (1.00, 3.00)	<0.001
**Primary outcome: mRS score**			
3-month mRS, median (IQR)	1.00 (0.00, 3.00)	0.00 (0.00, 1.00)	<0.001
3-month Poor outcome, *n* (%)	19.00 (33.90)	1.00 (2.30)	<0.001
**Other outcomes**			
Recurrent ischemic stroke, *n* (%)	1.00 (1.80)	2.00 (4.70)	0.41
Improved hemodynamics of diseased vessels, *n* (%)	35.00 (62.50)	23.00 (53.50)	0.37
Hemorrhage, *n* (%)	4 (7.14)	1 (2.32)	0.384

## Discussion

NLR is a composite marker of absolute peripheral neutrophil and lymphocyte counts and reflects the burden of inflammation (12). Previous studies showed that inflammation played an important role in the pathogenesis of arterial dissection and stroke (7, 18–20). In this study, we showed that high NLR was an independent predictor of 3-month poor outcome for AIS caused by CCAD. It was a convenient and simple indicator of inflammatory response after CCAD-induced AIS. To our knowledge, this study was the first time to analyze the relationship between NLR and 3-month outcome in CCAD-induced AIS patients.

Previous study showed that inflammation played an important role in the initiation and progression of ischemic cerebrovascular diseases (20). Therefore, NLR, the marker of inflammation, may also reflect the progress and prognosis of ischemic cerebrovascular diseases. Oz et al. (7) found that NLR at the time of admission was a predictor of the short-term outcome of patients with Stanford type aortic dissection. Kocaturk et al. (21) and Qun et al. (15) also confirmed that NLR can predict the 3-month prognosis of AIS. Considering that AIS caused by CCAD may have different inflammatory changes from AIS, we expanded the previous study by screening AIS patients with CCAD. We recruited 99 patients with AIS by CCAD as the research objects; however, we still found that NLR is a good predictor of poor outcome at 3 months. Interestingly, 79.8% of patients were male, showing a strong gender predisposition. In addition, for our total CCAD patients (*n* = 168 with or without AIS), male patients accounted for about 66.0%. In previous CCAD population-based studies, it seemed a slight gender predisposition favoring males (53–57%) (22). This may be due to the selection bias caused by small sample size in our study. However, male gender predisposition may be further examined in CCAD patients in future studies.

The increase in NLR indicates the suppression of lymphocytes or the excessive activation of neutrophils (12). After the occurrence of acute aortic dissection and AIS, systemic immune suppression may occur due to the brain's immune response (23–25), especially for T cells and natural killer cells in lymphocytes (26). The function of lymphocyte in ischemic brain injury and ischemic vascular endothelial is still controversial at present, but certain specific subtypes have been shown to play a protective role in the pathophysiology of cerebral ischemia (24, 26). Some studies suggested that lymphopenia was an early feature of stroke, which is a sign of persistent brain damage, stress response, and the greater possibility of infection (26). Acute stroke can trigger the reduction of regulatory T cells that suppress the inflammatory response to increase tissue damage (23, 24, 26, 27). We didn't find a correlation between low lymphocyte and 3-month poor outcome.

Considerable studies have demonstrated the damaging effects of neutrophils on vascular endothelial cells in arterial dissection and ischemic brain tissues (20, 28–32). Due to the similar pathogenesis, we speculate that neutrophils play an important role in the occurrence and development of AIS caused by CCAD. Infiltration of inflammatory cells can usually be found in the ischemic arterial walls of patients with CCAD and AIS (18, 28, 33). Peripheral neutrophils penetrate into the blood vessel walls and release vasoactive or cytotoxic media, including reactive oxygen species, proteases, matrix metalloproteinase (MMP), and cytokines, which may lead to the destruction of the extracellular matrix and the collapse of the vessel walls (32, 34). Our study found higher neutrophil count and higher leukocyte count baselines both correlate with 3-month poor outcome in the binary logistic regression analysis.

However, NLR reflects the balance of neutrophil and lymphocyte levels, which can comprehensively reflect the immune status, and the ratio may be more stable than a single parameter. In this study, we also used NIHSS at admission to analyze CCAD patients and found that the scale may not adequately capture all forms of functional change. The NIHSS has many advantages; however, it may miss some functional changes when used in place of neurological examination or blood parameters to measure improvement stroke (35, 36). Therefore, we collected both NIHSS score at admission and blood biochemical parameters to analyze their correlation with the prognosis of patients. Although statistical analysis showed that many factors, including NIHSS, were associated with prognosis, NLR was still independently associated with 3-month prognosis in binary regression analysis after adjusting for confounding factors. We took NLR = 2.97 as the cutoff value. The proportion of 3-month poor outcome in patients with high NLR was significantly higher than that in patients with low NLR, which was consistent with previous studies of a single ischemic stroke with a larger size of samples. In addition, we also found that the age and triglyceride level were associated with 3-month outcome. This may be related to the pathogenesis of CCAD. However, there was no significant difference between two groups in the improvement of the hemodynamics of the diseased vessels and the risk of recurrent ischemic stroke at 3 months.

A higher NLR level may indicate devascularization, early neurological deterioration and systemic immune dysfunction. These events increase the risk of death and poor outcome in AIS caused by CCAD. Generally, as an available clinical indicator, NLR has strong practicability for acute clinical decision-making and prognosis judgment.

There are also some limitations in our research. First, the number of cases of AIS caused by CCAD is relatively small. The insufficient sample size leads to weakened statistical strength of conclusions. Second, the study is observational in nature. We can't prove the causal relationship between NLR and 3-month adverse outcome. Third, our study included patients with anterior and posterior circulation dissections and did not explore the relationship between cerebral infarct volume and poor prognosis.

## Summary

The NLR level is related to the 3-month poor outcome of patients with AIS caused by CCAD. When NLR ≥ 2.97, the risk of poor outcome increases. This reliable and easy-to-use predictor could contribute to clinical treatment strategy design in patients with CCAD. Further studies should be performed to expand the sample size and investigate the relevant inflammation and immune pathways.

## Data Availability Statement

The original contributions generated for the study are included in the article/supplementary materials, further inquiries can be directed to the corresponding author/s.

## Ethics Statement

This study involving human participants were reviewed and approved by the Institutional Review Board of the First Affiliated Hospital of Soochow University. All patients provided their written informed consent.

## Consent for Publication

All the authors approved the manuscript and are consent for the submission.

## Author Contributions

GS, YY, ZC, and XX designed the study. GS, YY, ZC, SD, SH, YiqW, and YitW evaluated the subjects and collected the data. LY, BS, and YY analyzed the data. GS, YY, XY, and XX wrote the manuscript. All authors contributed to the article and approved the submitted version.

## Conflict of Interest

The authors declare that the research was conducted in the absence of any commercial or financial relationships that could be construed as a potential conflict of interest.
